# Experimental animal models of acute respiratory distress syndrome: one-hit and two-hit establishment application

**DOI:** 10.3389/fimmu.2025.1649074

**Published:** 2025-12-11

**Authors:** Yue Zhao, Xin Li, Yanhua Ou, Xiangran Cai, Weijian Huang, Shuhua He, Sisi Liang, Ning Wang, Linliang Song, Meixia Fang, Hatitao Niu, Jun He

**Affiliations:** 1 Institute of Laboratory Animal Science, Jinan University, Guangzhou, China; 2 Guangzhou Key Laboratory for Germ-free animals and Microbiota Application, Jinan University, Guangzhou, China; 3 Key Laboratory of Viral Pathogenesis and Infection Prevention and Control (Jinan University), Ministry of Education, Guangzhou, China; 4 The Sixth Affiliated Hospital of Jinan University, Dongguan, China; 5 The First Affiliated Hospital of Jinan University, Guangzhou, China

**Keywords:** acute respiratory distress syndrome (ARDS), animal model, modeling design and assessment, one-hit, two-hit

## Abstract

**Background:**

Acute respiratory distress syndrome (ARDS) is a complex syndrome with multiple risk factors that can lead to acute respiratory failure and, in turn, high morbidity and mortality. To clarify the syndrome’s underlying pathomechanisms and develop novel therapies, we have summarized and analyzed a series of chief cause-induced animal models of ARDS.

**Aim:**

Although various animal models have been developed to represent the traits of human ARDS based on clinical symptoms and the yardstick of positive clinical trials, each model has unique features that reflect only part of the characteristics modeled. In response, this review aims to investigate characteristics of ARDS in current animal models and offers new strategies and insights for developing animal models aimed at capturing the features of human ARDS.

**Conclusion:**

This review summarizes the physiological characteristics of animals used in models of ARDS and evaluates the advantages and disadvantages of the chief cause-induced models for modeling human ARDS in animals, for results that can inform the establishment, assessment, and experimental study of ARDS in animal models.

## 1 Introduction

Acute respiratory distress syndrome (ARDS) is an acute respiratory failure that occurs when exudative fluid accumulates in the pulmonary alveoli of critically ill patients due to increased alveolar–capillary membrane permeability and sustained inflammatory response. Manifesting as rapidly progressive dyspnea, tachypnea, and hypoxemia, ARDS typically emerges within a time frame ranging from a few hours to several days following the precipitating injury or infection (1, 2). In humans, ARDS is characterized by the acute onset of bilateral pulmonary infiltrates and severe hypoxemia with respiratory failure in the absence of cardiogenic pulmonary edema (3). The definition of ARDS, published by the American–European Consensus Conference in 1994 and revised as the Berlin definition in 2011, stipulates four criteria (1): emergence of clinical insult or onset of respiratory symptoms within a week (2), radiographic changes in the form of bilateral opacities (3), edema originating from noncardiogenic pulmonary failure, and (4) severity based on the PaO2/FiO2 ratio, with classifications of mild (i.e., PaO2/FiO2: 200–300), moderate (i.e., PaO2/FiO2: 100–200), and severe (i.e., PaO2/FiO2: ≤100) (3, 4). Nevertheless, limitations in the understanding of ARDS following the publication of the Berlin definition have surfaced, especially regarding noninvasive pulse oximetric methods, the syndrome’s Kigali modification, and the preclinical testing of novel therapeutics and interventions for ARDS (5, 6).

The various causes of ARDS are generally divided into direct pulmonary insults (e.g., pathogenic infection) and indirect insults to the lungs (e.g., sepsis) (7, 8). Because obtaining direct measurements of pathological lung tissue samples in most patients is unrealistic, the diagnosis of ARDS usually depends exclusively on clinical criteria (9). For that reason, various animals—mice, sheep, pigs, rabbits, and even tree shrews, among others—have been employed to investigate lung injury of ARDS. In that context, to elucidate the fundamental mechanisms of ARDS and explore therapeutic approaches to its treatment, using effective, reliable animal models that accurately mimic distinct features of human ARDS is crucial (10–14). To that end, the literature presents an array of values for physiological parameters in commonly used laboratory animal species, and those physiological parameters, as well as anatomical features, should be considered.

In this review, we list the specific physiological parameters of each animal model in order to compare their similarities and differences in pulmonary anatomy as well as physiology and thereby identify models that are ideal for the purpose (**Supplementary Material**, **Supplementary Table S1**). Among those parameters, animal size relates to the accuracy of physiological parameters such as arterial blood pressure and arterial oxygen partial pressure. Moreover, when collecting blood samples, large animals are more likely to obtain enough specimens to measure blood gas, plasma inflammatory factors, and neutrophils. Due to such differences between species, differences in their immune systems exist as well, including in pulmonary intravascular macrophages, nitric oxide production, and the presence of hyaline membranes, all of which determine unique species-specific manifestations of lung injury. Beyond that, in research on respiratory systems, aspects of handling, accessibility, costs, and standard reagents, among others, should be considered in features of animal models (**Supplementary Material**, **Supplementary Table S2**). Other considerations include the selection of animals that permit cost-effective housing and breeding as well as standard diagnostic procedures. Above all, researchers should thoroughly consider and evaluate the appropriateness of animals’ models and choose the most suitable species for each scientific question, while taking into account the unique advantages and disadvantages of each species. To that purpose, the various references available should be consulted to also review methodologies used and verify the specific species and, when appropriate, the animal model of ARDS used.

This review summarizes aspects of various species currently used to model ARDS using diverse modeling methods. In so doing, we aim to support research on choosing the most appropriate species, including small, medium, and large animals, for investigating ARDS in light of unique objectives. We also provide a comprehensive overview and analysis of the different models established using those species while considering distinct triggers of ARDS in each species.

## 2 Chief causes and modeling methods of ARDS

**Figure 1:**
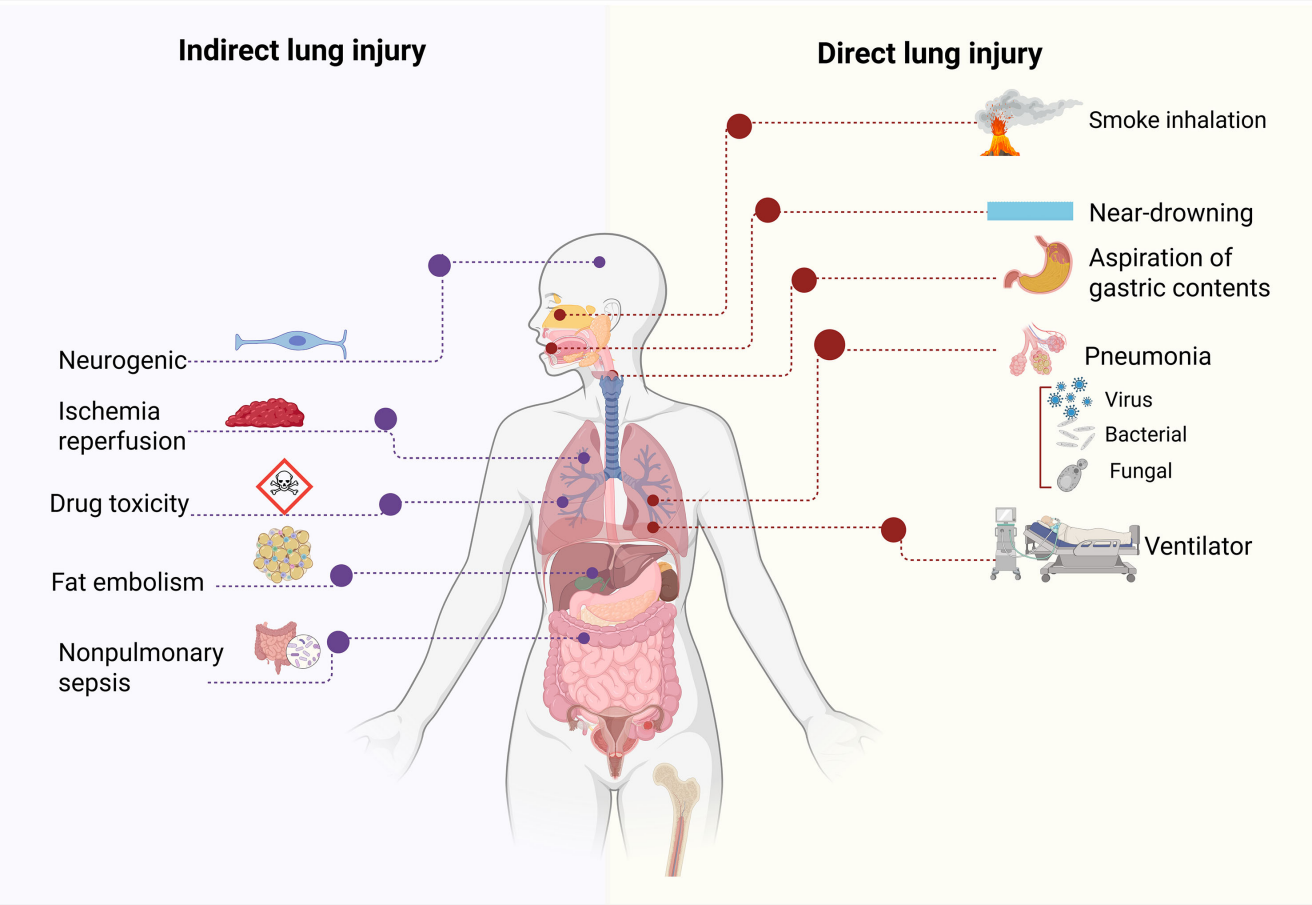
Causes of ARDS.

The various causes of ARDS are generally divided into direct pulmonary insults (e.g., pathogenic infection) and indirect insults to the lungs (e.g., sepsis) (**Supplementary Material**, **Supplementary Table S3**) (7, 8). The most common causes of human ARDS are pneumonia due to infectious (i.e., bacterial, viral, and fungal) triggers of the lung; nonpulmonary sepsis due to non-infectious triggers originating from sources such as the peritoneum, urinary tract, soft tissue, and skin; aspiration of gastric and/or oral and esophageal contents; and major trauma (e.g., blunt or penetrating injuries or burns) (**Figure 1**) (15–19).

Although the causes of ARDS are complex, the various triggers usually induce local or systemic inflammation, which results in severe hypoxemia, elevated edema, diffuse alveolar hemorrhage, and the formation of hyaline membranes (20–23). Less common scenarios associated with the development of ARDS include emergency transfusions, acute pancreatitis, drug overdose, near-drowning, hemorrhagic shock or reperfusion injury, and smoke inhalation (24–29). There are also several important risk factors for acute pneumonia, including excessive alcohol consumption, smoking, old age, and chronic lung disease (30, 31). In this review, we systematically analyze the causes associated with pneumonia or nonpulmonary sepsis in ARDS and their pathological characteristics (**Table 1**).

**Table 1:** Pathological characteristics of different animal models for ARDS.

Methods		Species	Histological characteristics							References
Modeling		Animals	Neutrophil accumulation in alveoli and interstitia	Hyaline membranes	Inflammatory exudation in alveoli	Thickened alveoli	Lung edema	Diffuse alveolar damage	Endothelial injury	
One-hit	LPS	Mouse	✓	−	✓	−	−	−	Less endothelial cell damage	(32, 33)
		Rat	✓	−	✓	✓	−	−	Severe congestion, hypertrophy	
		Sheep	✓	−	✓	−	−	✓	−	
	HCl	Mouse	✓	−	−	Thickened alveolar wall	✓	−	✓	(34–41)
		Rat	✓	−	✓	−	✓	−	−	
		Rabbit	✓	−	✓	Thickened alveolar wall	✓	−	Hemorrhage, swelling in microvascular endothelial and alveolar epithelial type I cells	
		Pig	✓	−	✓	Thickened alveolar wall	✓	−	Breeding	
	Hyperoxia	Mouse	✓	−	✓	−	✓	−	−	(42–47)
		Rat	✓	−	✓	Thickened alveolar septum	✓	−	✓	
	Bacteria	Mouse	✓	−	✓	−	✓	✓	−	(48–51)
		Rabbit	✓	−	−	−	✓	✓	−	
		Pig	−	−	✓	−	−	−	−	
	Virus	Mouse	✓	✓	✓	✓	✓	✓	−	(52–59)
		Nonhuman primates	✓	−	✓	✓	✓	−	−	
		Ferret	✓	−	✓	✓	−	−	−	
		Fruit bat	✓	−	−	−	✓	−	−	
		Hamster	✓	−	✓	−	−	−	−	
	Oleic acid	Mouse	✓	−	−	−	✓	−	−	(60–63)
		Rat	−	✓	−	✓	−	✓	−	
		Dog	✓	✓	✓	✓	✓	−	−	
		Pig	−	−	✓	−	✓	−	−	
	Smoke	Mouse	✓	−	✓	−	✓	−	−	(64–67)
		Rat	✓	−	✓	Thickened alveolar septum	✓	−	−	
		Pig	✓	−	✓	−	✓	−	−	
		Sheep	✓	−	−	−	✓	−	−	
	Surfactant depletion	Rabbit	✓	✓	✓	✓	−	−	−	(33, 67–69)
Two-hit	Hyperoxia + VILI	Mouse	✓	−	✓	−	−	−	−	(70, 71)
	Ischemia, reperfusion	Rat	✓	−	−	−	✓	−	−	(72–75)
		Rabbit	✓	−	−	−	✓	−	−	
		Pig	✓	−	−	✓	✓	−	−	
		Mouse	✓	−	−	−	✓	−	−	
	LPS + gastric or oleic acid	Pig	✓	−	✓	✓	✓	✓	✓	(76–79)
		Sheep	✓	−	✓	−	✓	✓	✓	
		Rabbit	−	✓	−	−	−	−	−	
	LPS + ventilator-induced	Mouse	✓	−	✓	✓	✓	−	−	(80–82)
		Rat	✓	−	✓	✓	✓	−	−	
		Rabbit	✓	−	✓	−	✓	−	−	
	Repeated saline lavage + ventilator	Pig	−	−	✓	✓	✓	−	−	(77, 83–85)
	HCl + VILI	Mouse	−	−	✓	−	✓	−	−	(86, 87)
		Rabbit	✓	−	✓	−	✓	−	−	
	HCl + biphasic positive airway pressure	Rabbit	✓	−	−	✓	✓	−	−	(88)
	HCl + LPS	Rat	✓	−	−	−	✓	−	−	(89)
	Smoke + burn	Sheep	✓	−	−	−	✓	−	✓	(90)

HCl, hydrochloric acid; LPS, lipopolysaccharide; VILI, ventilator-induced lung injury.

### 2.1 Causes of direct lung injury

According to the criteria of the American–European Consensus Conference, pneumonia, including nosocomial pneumonia, is the most frequent single cause of ARDS among critically ill patients, usually following diffuse alveolar damage (91). Typically triggered by insults induced by chemical agents, infection, smoke, or mechanical injury within one-hit or two-hit direct administration, cases of ARDS involve the alveolar–capillary barrier’s rapid aggravation of the pathophysiology of lung injury and, in turn, symptoms of severe hypoxemia that mimic the properties of human ARDS (92).

#### 2.1.1 One-hit direct insult in animal models

**Figure 2:**
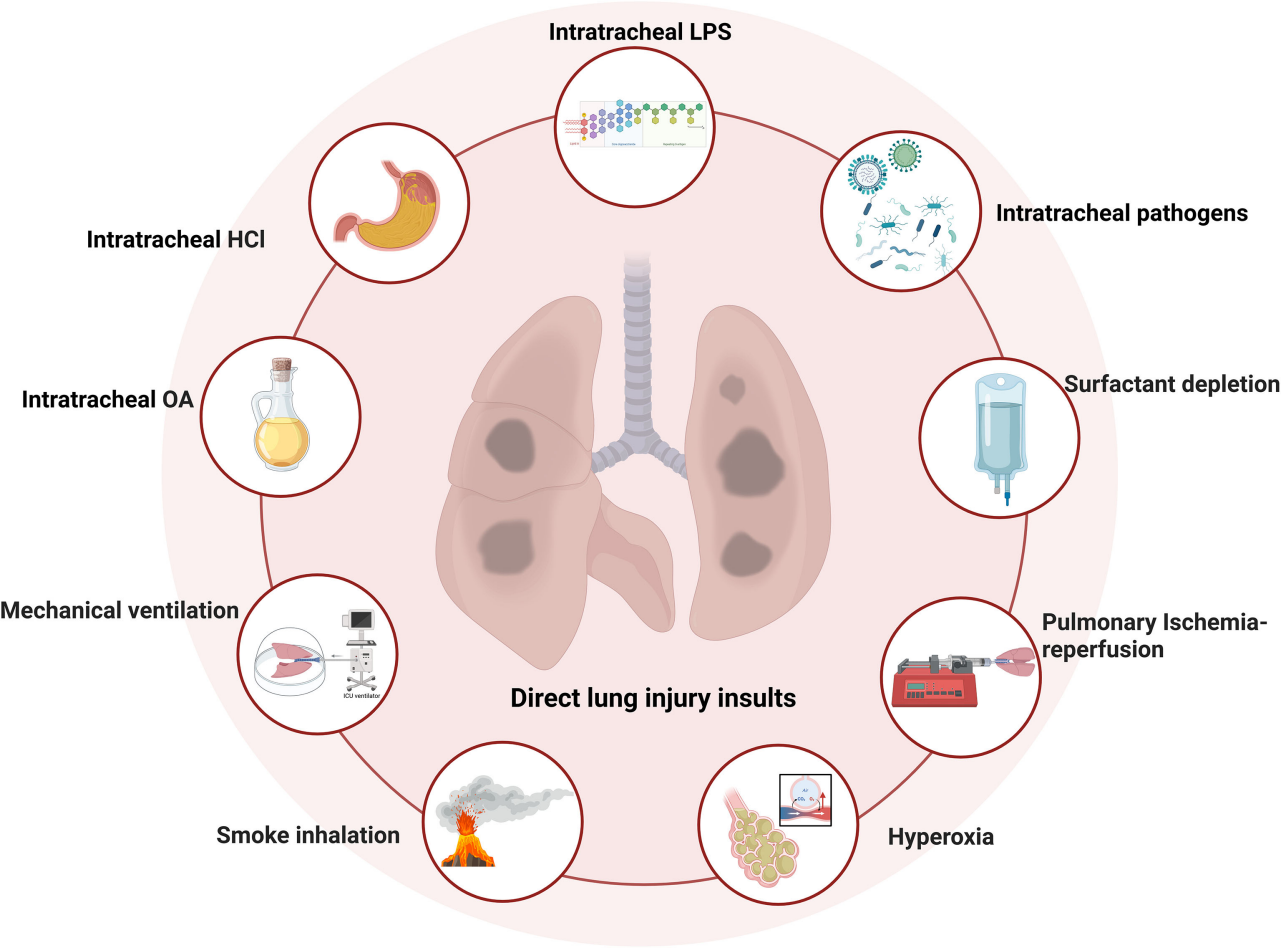
One-hit direct lung injury induced by severe pneumonia in animal models (created with BioRender, https://app.biorender.com/).

The ideal strategy for establishing ARDS animal models is to induce severe pneumonia via one, two, or multiple combinations. One-hit intratracheal administration with a high dose of LPS, Gram-negative bacteria, viruses, acid (e.g., hydrochloric acid or oleic acid), or smoke can cause severe lung injury as well as neutrophil infiltration, permeability edema, and rapid immune response that mimics a direct response to ARDS (**Figure 2**) (93–96).

##### 2.1.1.1 Intratracheal LPS

LPS modeling, which is widely used in models of small animals (e.g., mice and rats) and medium-sized animals (e.g., rabbits), can imitate acute pulmonary inflammation and a damaged alveoli-capillary barrier and exhibits many mechanisms in the early acute phase, primarily the first 24 h (97–100). Whether observed from histological images or other methods of detection, LPS-induced immune responses resemble those in humans. However, lung injury and physiological data observed in such animal models depend on early detection, which suggests that researchers should use accurate times in their experimental trials. Even so, during investigations of the pathogenesis of ARDS and drug protection in animal models of LPS-induced ARDS, the endpoint has extended to 48–72 h (101). Researchers have also established an ARDS model with the tree shrew, a prosimian species, via one-hit intratracheal LPS and found the comparable advantages. Although that modeling method has been extensively published, physiological data about the early endpoint in animal models induced by high doses of LPS are more closely resemble toxicity-related data.

##### 2.1.1.2 Oleic acid and hydrochloric acid

The symptoms of severe hypoxemia in ARDS models are directly caused by the administration of oleic acid, hydrochloric acid (HCl), bleomycin, or the lavaging of the lungs in animal models. Similar to fatty acids released from fractured bones, those stimuli appear to damage endothelial cells and be responsible for high mortality due to hemodynamic instability (102). Given the economic advantages and applicability of these reagents, it has primarily been used in large animal models for ventilation strategies and lung mechanics. Such models can achieve a low PaO2/FiO2 ratio and typical bilaterally diffuse infiltrates observed in large animal models (103). For example, as shown in a goat model, lung injury induced by oleic acid can lead to severely impaired gas exchange, the deterioration of lung mechanics, and the disruption of the alveolar–capillary barrier (104). In addition, the histological analysis of the lungs of ARDS in animal models has also revealed the infiltration of inflammatory cells, pulmonary edema, and microvascular injury, which could serve as a good models for study of pathophysiology for the syndrome (105). The intratracheal instillation of HCl to mimic direct ARDS via the bronchial aspiration of gastric contents has been widely used in rabbit models (106). Although exhibited decreased arterial oxygen pressure has been observed in small model (rabbit model) induced by HCl, only 10 articles in the database of the National Center for Biotechnology Information (NCBI) pertain to that dynamic.

##### 2.1.1.3 Ventilator-induced ARDS

Mechanical ventilation, first described in the mid-18th century, used animal models that is induced by clinical applications performed in humans and associated with lung injury (107–110). The ventilator-induced lung injury (VILI) model, the most translatable finding in animal models, is influenced by the animal’s size, whether the thorax is open or closed, and whether extrinsic positive end-expiratory pressure is used. Various animal models have been employed, including large mammals (e.g., pigs and sheep) and small rodents (e.g., mice and rats), as well as rabbits (111–114). However, small animals are prone to temperature variability, hemodynamic instability, and limitations of invasive monitoring, which cause unrealistically extended periods of supportive care (115). Therefore, due to its similarity in respiratory anatomy and physiology to humans, the porcine model is usually chosen for one-hit VILI models (116). Unfortunately, the model’s characteristics often last only a few hours. Such short-term models usually induce mild inflammation without any loss of lung function, which limits understandings of the process of ARDS induced by VILI (117, 118).

##### 2.1.1.4 Smoke inhalation

Animal models with ARDS induced by smoke inhalation typically use large animals, although small animals can be induced as well, to evaluate clinical symptoms, pathological lesions, and pathogeneses associated with the clinical identification of indicators of ARDS among clinical patients (119). For instance, in pigs with smoke inhalation-induced lung injury, the PaO2/FiO2 ratio is approximately 208 (50%), while bilateral diffuse infiltrates have been visible on chest X-ray (120). Sheep are another commonly used animal model for ARDS caused by smoke inhalation, which may be a valuable model for studies on pneumonia (121). Although smoke inhalation amounts to genuine intoxication with ARDS in clinical patients, many mechanisms are involved in ventilator-induced lung injury with different time points from clinical manifestations. However, due to disease kinetics, the rapid instillation of high-dose smoke, similar to the rapid infusion of high-pathogens, usually represents a model for intoxication, not pneumonia (122).

##### 2.1.1.5 Intratracheal pathogens

Infectious pneumonia is categorized as viral pneumonia caused by influenza viruses (e.g., H5N1 and H1N1 2009) or coronaviruses (e.g., SARS-CoV-2); bacterial pneumonia caused by Streptococcus pneumoniae or Pseudomonas aeruginosa; or parasitic pneumonia caused by Plasmodium falciparum (53, 123–126). Animal models exhibiting the direct infection of the respiratory tract by pathogens are typically used to study the pathogenesis of pathogens and screening with antigenic drugs, for most models can reveal typical pathological features and immune system responses. Recently, the pandemic caused by SARS-CoV-2 infection has been associated with ARDS in clinical manifestations, sometimes in fatal cases (127). The outbreak of the disease has caused a significant rise in ARDS and heightened public awareness of the syndrome. Partly in response, scientists have developed a genetically modified mouse model expressing the human ACE2 receptor, one that can exhibit severe pneumonia, alveolar necrosis, significant lymphopenia, and neutrophilia in peripheral blood after insult with SARS-CoV-2 (128–130). Nonhuman primates offer another well-established model of infection with SARS-CoV-2, SARS-CoV, and H5N1, which are invariably accompanied by strong pro-inflammatory responses, inflammatory cytokine response, and hyaline membrane formation, all of which are rarely found in rodent models (131, 132). Other commonly used animal models, including hamsters and ferrets, are widely studied to investigate highly pathogenic respiratory viruses, which can mimic one or more characteristics of human ARDS (52, 133–135). However, for biosafety’s sake, it is necessary to conduct experimentation in biosafe laboratories with pathogen limits, and laboratory personnel need to undergo rigorous training to ensure such safety (136–139).

Other intratracheal pathogens are bacteria (e.g., S. pneumoniae and Pseudomonas aeruginosa), which in animals such as mice, rats, and pigs can induce pneumonia within 7 d. Even so, most animals eventually recover (140–142). For a good model of pneumonia-induced ARDS, typical pathological characteristics should be observed during the induction period, including neutrophil infiltration in interstitia and alveoli and epithelial cell injury. The duration of the animal model’s construction depends on bacterial load and activity, which strictly require certain technical operations from researchers.

##### 2.1.1.6 Surfactant depletion

Another direct cause of lung injury is the reduction in levels of pulmonary surfactant, which leads to an increase in surface tension and a decrease in lung compliance during respiration (143). Saline lavage is the most common method that leads to surfactant depletion; it has been widely studied in the context of treating ARDS induced by mechanical ventilation, and pathological and physiological research on the process of ARDS (144). Large and medium-sized animal models (e.g., rabbit, sheep, and pig) are frequently applied to research on lung injury induced by saline lavage with surfactant depletion (145–148). In anesthetized pigs subjected to repeated lung lavages with warmed 0.9% saline (50 mL/kg body weight), severe lung injury has been induced and led to a reproducible deterioration in pulmonary gas exchange and hemodynamics, accompanied by the disadvantageously high recruitability of atelectatic lung tissue (149). Similarly, intratracheally instilled surfactant (100 mg/kg) in newborn piglets has been shown to induce significantly decreased alveolar–arterial oxygen and pulmonary histologic damage, which suggests that such models can be expected to become protective models for drug screening (150).

##### 2.1.1.7 Pulmonary ischemia and reperfusion

Lung injuries induced by pulmonary ischemia and reperfusion (IR) are the most critical mechanism responsible for ARDS in patients following esophagectomy and its leading cause following cardiothoracic surgeries, which results in severe lung dysfunction (151, 152). Generally, lung ischemia is induced by either anoxia or lack of ventilation; thus, the standard methods for blocking blood flow are cross-clamping the vessels via arterial forceps and ligatures and using balloon occluders in the lungs (151). In animals, the lungs of male Sprague–Dawley rats have been flush-perfused with a modified natural bovine surfactant at a dosage of 50 mg/kg body weight, which significantly lessened intra-alveolar edema formation and the development of atelectasis. Pigs, due to their physiological and anatomic similarities to humans, have been identified as suitable models for studying ARDS as well as IR injury, which is the most critical mechanism responsible for developing ARDS in patients after esophagectomy (151).

##### 2.1.1.8 Hyperoxia

Hyperoxia, characterized by excess oxygen in tissues and organs, can induce diffuse pulmonary injuries, vascular leakage, excessive inflammation, and pulmonary edema (153). In research conducted on mice, both CCR2+/+ mice and CCR2−/− mice were exposed to 85% O2, and all died within 6 d. Neutrophils, lymphocytes, and macrophages were significantly elevated, while severe alveolar hemorrhage with a slight thickening of alveolar walls and focal inflammatory cell infiltration was observed in both groups (154). In other research, female C57BL/6J mice were exposed to 90% O2 and exhibited inflammatory infiltrations of neutrophils and other factors, along with edema, thickening of the alveolar walls, and elevated levels of indicators of oxidative stress (153). Similarly, C57BL/6J mice exposed to 95% O2 exhibited a large number of proinflammatory cytokines (e.g., TNF-α, IL-6, and IFN-γ) and increased levels of macrophages and neutrophils infiltrating the lung tissue of mice (155, 156). Despite those findings, the precise mechanism underlying hyperoxia-induced ARDS remains incompletely understood, and effective therapies have not yet been developed.

#### 2.1.2 Two-hit animal models

Patients who suffer from lung injury commonly experience two or multiple hits (i.e., “two-hit” and “multiple-hit,” respectively), which prolongs the immunological response to injury and manifests in clinically significant lung injury (157). In such cases, the singular cause of ARDS models is limited because those models fail to capture the multifactorial pathobiology of clinical ARDS. To better mimic clinical scenarios, two-hit animal models have been established, which are more technically challenging and have been proposed for immune responses with apparent success.

##### 2.1.2.1 Intratracheal LPS or HCl application with mechanical ventilation

Considering mechanical ventilation’s critical role in the care of critically ill patients, serious lung injury is usually initiated by intratracheal LPS application or subacute acid aspiration, with VILI often emerging soon after. Therefore, combining two methods to establish ARDS animal models may closely replicate traits of clinical patients induced by complex causes, as shown in many articles examining various animals. In one study, mice that received LPS 24 h before ventilation with ARDS displayed weight loss, less activity, piloerection, and tachypnea during the first 48 h, consistent with clinical signs of ARDS (158). Similarly, mice that received intratracheal LPS recovered for 20 h, then underwent mechanical ventilation for 4 h, and showed significantly higher inflammatory response levels of neutrophilic infiltration, interstitial edema, and massive alveolar wall damage in the histologic sections of lungs (159). Instead of LPS, HCl was intratracheally administered into the right bronchus of mice, which were subjected to mechanical ventilation after 3 h of surfactant administration; the arterial blood pressure of those mice reached 40 mm Hg, which led to a slow death and the release of inflammatory mediators (i.e., IL-1β and TNF-α) (160). That modeling method is also achievable in rat models, though the duration of the human endpoint cannot be mimicked (161).

The combined method with ventilators in large animal models can quickly induce expected symptoms, including extended life endpoints, hypoxemia, and other symptoms rarely observed in small rodents when severe lung injury is simulated (162–164). A model of highly severe recoverable ARDS was induced in pigs using a two-hit model of lung injury involving 0.9% warm saline lavage and high-volume ventilation (<100 mmHg); however, it lasted only several hours (162). More recently, a multidrug-resistant Pseudomonas aeruginosa strain was intratracheally a multidrug-resistant Pseudomonas aeruginosa strain in pigs after VILI was applied for 3 h, and the ratio of PaO2/FiO2 reached 83 ± 5.45 mmHg, with reduced static compliance, increased pulmonary permeability, and a duration of diffuse alveolar damage exceeding 40 h accompanied by high mortality (165). That modeling method has been used in pigs and sheep, and most associated research has focused on the therapeutic effect of ventilation and the long process of altered breathing. Although the model can present clinical symptoms, especially prolonged symptoms as defined by the new Berlin definition of ARDS, the duration of the symptoms is difficult to control, which poses significant difficulties for studying the syndrome’s pathogenesis.

### 2.2 Nonpulmonary sepsis

Nonpulmonary triggers can indirectly induce systemic inflammation, including pancreatitis, aspiration of gastric contents, and severe traumatic injuries with shock and multiple transfusions.

#### 2.2.1 Acute pancreatitis

Acute pancreatitis is a crucial gastrointestinal cause of hospital admissions in both humans and dogs. In severe cases of acute pancreatitis, a systemic inflammatory response syndrome, is responsible for up to 60% of mortality. However, because of the condition’s short duration and uncontrollability, it is rarely used to study ARDS (166).

#### 2.2.2 Mesenteric ischemia and reperfusion

Ischemia, associated with anoxia and the absence of nutrient supply, typically causes oxidative damage to tissues, the release of inflammatory mediators, and the influx of inflammatory factors to local and remote organs. In past research, IR has been induced in mice via a 45 min occlusion of the mesenteric artery, followed by reperfusion lasting 2 h. Ultimately, it caused increased myeloperoxidase expression and neutrophils in lung tissue. That modeling approach rapidly produces anticipated symptoms and has a high success rate associated with a significant decrease in arterial oxygenation level at 24 h post-administration (167). In other research, ventilated pigs have been subjected to experimental sepsis via the placement of a peritoneal fecal clot and IR by clamping the superior mesenteric artery for 30 min to achieve more typical clinical symptoms. The markedly decreased oxygen index and P/F ratio, as well as numerous inflammatory, variable physiologic, and blood chemical manifestations, were observed (168). That type of modeling method is rarely showcased in literature in the NCBI database, and surgical modeling may have higher requirements for the operator.

## 3 Summary

Human ARDS is a serious complication of systemic severe inflammatory response syndrome caused by various severe injuries to the lung, including sepsis, trauma, pneumonia, and smoke inhalation injury, and diffuse alveolar damage (DAD) is a histological manifestation of ARDS. Many neutrophils and macrophages, which are inflammatory cells that play a crucial regulatory role in innate immunity, infiltrate the lung tissue induced by DAD. Although the causes of ARDS are complex, the inflammatory response plays a key role in all animal models of ARDS—for example, infiltration of neutrophils, dysregulated immune-inflammatory responses, and abnormal activation of macrophages. By inhibiting the production of an inflammatory response, immune regulation can relieve the inflammatory cells in ARDS. Here, we summarized key immune cell types (such as pulmonary intravascular macrophages, neutrophils, T cells, and interleukin) and pulmonary pathology of a series of ARDS animals (**Table 2**).

**Table 2:** Pulmonary immune and pathology.

	Modeling method	Human	Nonhuman primate	Pig	Goat	Sheep	Dog	Rabbit	Guinea pig	Tree shrew	Rat	Mouse
Pulmonary immune	Neutrophils (×10^3^/mm^3^)	2.45-5.25	4.86-7.17	3.1~5	2.07-2.39	5.64-6.77	6.0~12.5	2.5~6.0	2.0~7.0	0.24~0.98	1.1~6.0	0.7~4.0
	Pulmonary intravascular macrophages	No	No	Yes	Yes	Yes	No	No	No	−	No	No
	Hyaline membrane	Yes	Yes	Yes	Yes	Yes	Yes	Yes	Yes	Yes	Yes	Yes
	Nitric oxide production	Low	Low	Middle	Middle	Middle	Middle	Middle	Middle	−	High	High
	T cells	CD4^+^:Th1, Th2, Th17, Tfh, Treg CD8^+^ CD3^+^ CD14^+^ γδ T NK	γδ T	CD3^+^ CD14^+^ γδ T	−	CD3^+^	−	−	−	−	CD4^+^:Th17, Treg CD8^+^ CD3^+^	CD4^+^:Th1, Th17, Treg CD8^+^ CD3^+^ γδ T17 cells NK
	Interleukin	IL-1, IL-2, L-3, IL-4, IL-5, IL-6, IL-8, IL-10, IL-1β, IL-15, IL-18, IL-27, IL-32, IL-33, IL-35	IL-1β, IL-6, IL-8	IL-1, IL-3, IL-5, IL-6, IL-8	IL-6, IL-8	IL-1, IL-6, IL-8	IL-1β, IL-6, IL-8, IL-10, IL-12p70	IL-1, IL-6, IL-8, IL-10, IL-12, IL-13	IL-4, IL-6, IL-8, IL-12, IL-13	IL-6, IL-8, IL-8, IL-10, IL-17A	IL-1, IL-2, IL-6, IL-8, IL-10, IL-17A, IL-18	IL-1β, IL-6, IL-8, IL-10, IL-17A, IL-33
		TNF-α	CXCL1, CXCL2, CCL3, IFN-β	TNF-α, IFN-γ	−	TNF-α	TNF-α, IFN-β	TNF-α, NF-κB	−	−	TNF-α, IFN-γ	TNF-α, IFN-γ
Pulmonary pathology	Diffuse alveolar damage	✓	−	✓	−	−	✓	−	−	✓	−	−
	Alveolar epithelial injury	✓	✓	✓	✓	−	✓	✓	✓	✓	✓	✓
	Lung endothelial injury	✓	✓	✓	✓	−	✓	✓	✓	✓	✓	✓
Hypoxemia symptoms		−	−	−	−	−	−	−	−	✓	−	−
References		(169–178)	(131, 179)	(180–183)	(184)	(185, 186)	(187–189)	(190)	(191, 192)	(193, 194)	(195–198)	(101, 128, 178, 199–203)

*Note: － not detected.* As an innate immune cell, macrophages play a key role in the inflammatory response. Pulmonary intravascular macrophages (PIMs) are a potential reservoir of viral and other infectious agents in inflammatory response of lungs, thus contributing to the pathogenic burden in the lung (204). PIMs are constitutively found in species such as pigs, sheep, goat, cats, and tree shrews and can be induced in species such as humans and rats, which generally lack them. These immunological differences determined that animals within PIMs are very susceptible to lung inflammation, but it is difficult to cause any loss of lung functions in large animals. However, one significant unexplained difference between human ARDS and many other models is that all the animals that survive the challenge recover completely. In contrast, this is different from ARDS patients, where it is still a mystery why some patients fail to heal, which is one of the central questions in ARDS. Notably, lung inflammation occurs relatively easily, which lets us realize that not every lung inflammation is ARDS. Another critical issue is that a few ARDS patients die of lung failure alone, and the death occurs due to multiple organ failure (205). In addition, diffuse lung inflammation induces DAD in humans, activating and dysregulating systemic inflammation and coagulation. However, treating inflammatory injuries is a coordinated process; treatment needs to be tailored to the problem instead of simply treating the causes in animal models.

## 4 Conclusion

Due to the difficulties in collecting clinical case data, especially pathological data, animal models have played a significant role in studying ALI. However, the replication of animal models has always been a challenge in research on ARDS. Different species should be considered when studying ARDS depending on the scientific question being investigated. In experiments using the same administration route, different animal species have manifested varying degrees of lung injury caused by different drugs, and even individuals of the same species but different ages have not reacted the same. Those complexities have to be considered when choosing the appropriate ALI/ARDS model. Due to the practical reasons mentioned, mice and rats are the preferred choice for most researchers, especially in studying signaling pathways, immune responses, and pathogenic processes, to explore the mechanism or identify potential therapeutic targets. Although the clinical symptoms of ARDS are not noticeable compared with humans, and the syndrome’s survival time is extremely short, transgenic mice are the first choice when studying ARDS’s pathogenesis, drug screening models, and related gene functions of ARDS. However, anatomical and immune differences and unobservable clinical symptoms between small rodents and humans require using larger animals for experimental validation. In particular, considering the characteristics of commonly used species, especially the anatomical features of the lungs, and the different modeling methods, we have summarized the clinical and pathological characteristics of each species under various modeling methods (**Table 1**). In this review, primary different modeling methods have been shown to involve various experimental procedures depending on the initial research interests and to have inevitable advantages and disadvantages (**Table 3**).

**Table 3:** Pathological characteristics of ARDS in different animal models.

Animal models	Model types		References
	Advantages	Limitations	
Mouse and rat	• Cost-effectiveness • Ease of handling • Suitability for large-scale studies • Ability to construct genetically engineered mice using multiple methods • Activation of innate immune response • Commercial (re)agents available and comprehensive	**Endpoint:** Animals may die of shock, not ARDS; survivors of initial phase recover completely. Models: <72 hours **Hypoxemia:** Difficult to induce **Severity of lung injury:** Little insight **Hyaline membranes:** Difficult to obtain **Inbred animals:** Not representative **Higher cytokine responses:** Higher in animals than humans	(72, 206, 207)
Tree shrew	• Cost-effectiveness • Activation of innate immune response • Commercial (re)agents available and comprehensive	**Endpoint:** Survivors of the initial phase recover completely. Models: >72 hours **Hypoxemia:** Easy induced **Severity of lung injury:** Little insight **Hyaline membranes:** Difficult to obtain **Inbred animals:** Not representative **Higher cytokine responses:** Higher in animals than humans	(208–210)
Rabbit	• Inflammatory exudation in alveoli	**Endpoint:** Animals may die of respiratory failure, not ARDS. **Hypoxemia:** Difficult to induce **Severity of lung injury:** Little insight **Hyaline membranes:** Difficult to obtain **Inbred animals:** Available but less commonly used than mouse and rat models **Higher cytokine responses:** rabbits differ from humans in terms of lung defense immune system, as the lack PIM in their bodies, and those macrophages can reduce endotoxin mediated lung injury responses	(72, 211)
Sheep	• Inflammatory exudation in alveoli	**Endpoint:** Animals may die of respiratory failure or multiple organ dysfunction syndrome. Models: >72 hours **Hypoxemia:** Easy induced **Severity of lung injury:** Mild or severe depending on dose of reagent **Hyaline membranes:** available **Inbred animals:** non-inbred line **Higher cytokine responses:** cannot measure cytokines in lymphatic fluid and determine left atrial pressure	(212–216)
Pig	• Pulmonary edema	**Endpoint:** Animals may die from severe respiratory failure, persistent hypoxemia, or refractory acidosis. Models: >72 hours **Hypoxemia:** Easy induced **Severity of lung injury:** Mild or severe depending on dose of reagent **Hyaline membranes:** Available **Inbred animals:** Non-inbred line **Higher cytokine responses:** Higher in animals than humans	(204, 217–220)

In addition, we summarize the relevance of lung features between humans and ARDS animal models based on the one-hit method. Neutrophil infiltration, epithelial cell damage, alveolar wall thickening is usually observed through intratracheal LPS, pathogens, or chemical reagents on animals, which will help understanding the pathophysiological mechanisms involved in the early process of human ARDS. While hyperoxia isn’t really likely in adult patients, ventilator, surfactant depletion and smoke inhalation is often a cause of lung injury, which is prefer to large animal models to treat the systemic inflammation, refine the mechanism and improve the novel therapeutic method (**Table 4**).

**Table 4:** Advantages and disadvantages of each ARDS modeling method.

Model type		Lung features mimic humans	Advantages	Disadvantages	Clinical relevance
Direct lung injury insults	Intratracheal endotoxin (LPS)	Neutrophil infiltration, alveolar edema, epithelial cell damage, alveolar wall thickening, early increase in collagen fiber	Potent activator of innate immune response, reproducibility, ease of administration	Less endothelial cell damage, prefer small animals, significant interspecific differences, variable endotoxin purity	Treatment of lung inflammation Mechanism research
	Viruses	Neutrophil infiltration, epithelial cell damage, alveolar wall thickening	A good model for viral pneumonia induced ARDS	Technical difficulties due to virus culture and personnel operations, high dose cause die of shock, biosafety should be concerned	Treatment of lung inflammation Mechanism research
	Bacteria	Neutrophil infiltration, epithelial cell damage, alveolar wall thickening, short-term recovery	A good model for pneumonia induced ARDS	Technical difficulties due to bacteria culture and personnel operations, biosafety should be concerned	Treatment of lung inflammation Mechanism research
	Hydrochloric acid	Alveolar epithelium injury, hemorrhage, necrosis and apoptosis of epithelial cells, alveolar edema, modest neutrophil infiltration	A good model for study of physiological impact of ARDS and ventilator-induced ARDS	Prefer middle or large animals, noninjurious dose is limited	Treatment of lung inflammation Mechanism research
	Hyperoxia	Severe alveolar haemorrhage, alveolar edema, Macrophages and neutrophilic infiltration, focal inflammatory cell infiltration	A good model for hyperoxia induced ARDS	Neutrophilia in interstitium and alveoli less marked than in human ARDS	Treatment of lung inflammation Mechanism research
	Ventilator-induced	Epithelial cell damage, interstitial edema, alveolar capillary damage	A good model for study of mechanical ventilation in clinically	Complex model, certain requirements for operators, such as deep anesthesia, mechanical ventilation; difficult to induce substantial lung injury without other stimulus	Treatment of systemic inflammation refine the mechanism Novel therapeutic method
	Surfactant depletion, i.e. Saline repeated lavage	Very few neutrophils, alveolar collapse, less tissue damage	A good model for study of detection ventilation in clinically	Complex model, certain requirements for operators, such as deep anesthesia, mechanical ventilation	Treatment of systemic inflammation refine the mechanism Novel therapeutic method
	Lung ischemia reperfusion	Increased alveolar-capillary damage with neutrophil infiltration, hemorrhage, interstitial and alveolar edema	A good model for study of ischemia reperfusion of human ARDS in clinically	Complex model, certain requirements for operators, such as deep anesthesia, mechanical ventilation	Treatment of systemic inflammation refine the mechanism Novel therapeutic method
	Smoke inhalation	Diffuse alveolar damage	A good model for study of smoke inhalation of human ARDS in clinically	Less complex model, certain requirements for operators, such as anesthesia, smoke operation	Treatment of systemic inflammation refine the mechanism Novel therapeutic method

For instance, small animal models are tractable, which can accommodate mechanisms of disease and allow exploration for therapeutic designs but cannot replicate all the clinical characteristics of ARDS. Large animal models, by comparison, can reproduce clinical features of ARDS and therefore serve as important models to preclinical treatments; however, they are less malleable in mechanistic studies. On top of that, hypoxemia, as the primary criterion of physiological dysfunction in humans, ranks among the biggest technical challenges in models of ARDS in small animals, except tree shrews, but can be actualized in models of large animals. By contrast, rodent models may support the goals of investigating genetic variants and predisposing conditions more readily than large animal models can. Because the animal models currently available cannot fully address human ALI, models need to accommodate at least three of the four domains to qualify as experimental ALI. In this review, we have provided practical, efficient methods for laboratories that lack the time and/or resources to develop complex models that are particularly suitable for studying specific disease processes in ARDS.

When selecting animal models to investigate the pathogenesis of ARDS, the following factors should be considered for selection. First, it is crucial to determine the endpoint in experimental studies. The data collected during experiments usually depend on the design time point or endpoint, which should be listed and clearly defined in the experimental trial design in order to ensure accurate timing. Many experimental endpoints are relative, including the original experiment involving neutrophil fraction in bronchoalveolar lavage cells or slight lung injury with inconspicuous symptoms evaluated by Evans blue, most of which focused on the early immune responses occurring within 24–48 h post-modeling. When trials involve the P/F ratio, wet/dry ratio, or compliance, endpoints should be prolonged, but small animals always die within 72 h in most studies. Large animal models could solve that problem, for the duration of ARDS exceeds 72 h, thereby allowing the detection of predictable clinical symptoms and physiological and biochemical indicators.

Second, the disease’s characteristics are a crucial aspect to consider. In high doses of pathogen-induced pneumonia and sepsis trials, there is a gradual growth pathogen with higher atypical, concomitant immune responses than in human patients. That circumstance may explain why anti-cytokine treatments have failed in humans. The slow administration of pathogen is the preferred modeling method, especially when combined with two-hit or even multiple-hit modeling methods, which can slowly induce lung inflammation and gradually achieve severe lung injury. Animal lungs are strong, redundant, and more importantly, hypoxemia is challenging to induce in healthy animals, especially in small animals, whereas hypoxemia is temporarily observed in large animal models.

Last, ARDS can be a respiratory failure, and the role of experimentation and pharmacotherapy in the treatment strategies of ARDS is minimal. The yardstick for positive clinical trials remains lacking, which may hamper researchers from achieving the requirements for a clinically relevant ARDS model. In fact, most hypotheses regarding human ARDS can be directly assessed in an extensive spectrum of animal models, which can be expected to lead to more acceptable results because of the intact in vivo environment. Although small and medium animals, including mice, rats, tree shrews, and rabbits, are simple, economical, and easy to use, their relevance to the clinic is limited. Conversely, large animals such as dogs, sheep, nonhuman primates, and pigs are more complex, expensive, and complicated to handle. Their advantage lies in their simulation of clinical cases, owing to their similarities with humans, especially in parameter analysis and multiple blood tests. The definition of ARDS has evolved through multiple updates to reflect new clinical insights and practical considerations, meaning patient-tailored strategies hold promise.

Overall, animal models remain helpful and will continue to provide valuable insights into the underlying pathogenesis, progression, and treatment of ARDS, as well as approaches for therapeutic regulation. Animal models should achieve at least three of four domains of ARDS in most mechanistic studies. However, models are recommended to fulfill all four domains in the preclinical testing of new interventions and therapeutics. The causes of human ARDS are complex, which makes it difficult for single animal models to perfectly mimic the symptoms of human ARDS. Researchers should rely on their specific scientific questions in choosing animal models; for example, small models are usually selected for studies on preliminary mechanisms; large animals tend to be evaluated for hypoxemia-related therapeutic strategies and the effectiveness of treatments; and multiple-species animal models should be considered for more reliable experimental results before human clinical trials. With the development of genetic research technology, it is also becoming common to use genetically modified animals for further research on ARDS and its underlying pathogenesis.
